# Cure MAPT FTD: MAPT Families Fighting for a Future

**DOI:** 10.1002/alz70855_105635

**Published:** 2025-12-24

**Authors:** Linde L Jacobs

**Affiliations:** ^1^ Cure MAPT FTD, Denver, CO, USA

## Abstract

**Background:**

Frontotemporal dementia is the most common form of dementia impacting those under the age of 60. It is estimated that 30% of affected persons have a genetic predisposition to this disease, with mutations in the genes encoding progranulin (*GRN*), chromosome 9 open reading frame 72(*C9orf72*), and microtubule associated protein tau (*MAPT*). Mutations in *MAPT* were discovered in 1998, yet to date, there have been no therapies or multisite clinical trials available to families.

**Method:**

To coordinate the sharing of information and advocacy efforts, a group of family kindreds impacted by *MAPT* created a non‐profit organization and database for families in November 2023.

**Result:**

Spanning over 6 generations across 10 countries in North America, South America, Europe, Australia and Asia we have organized 42 family kindreds with members living or deceased with symptomatic disease, pre‐symptomatic positive carriers, tested negative/non‐carriers, and at risk or untested family members which has a touch of over 500 individuals.

**Conclusion:**

We are aware of the burden of this disease on patients and families and the lack of resources to efficiently diagnose and treat FTD. We are working collaboratively with other genetic FTD organizations and institutions to further reach *MAPT* families in an international effort to provide support for *MAPT* families and gene carriers, identify areas for advocacy, promote research, and to contribute to future therapeutics. Fueled by the past trauma of watching loved ones succumb to FTD, terrified of how FTD will dictate the future, and fighting for those whose minds are currently failing them.

